# Ineffective Erythropoiesis Markers in β-Thalassemia: A Systematic Review

**DOI:** 10.3390/jcm15010308

**Published:** 2025-12-31

**Authors:** Kartika Prahasanti, Ami Ashariati, Lilik Herawati, Pradana Zaky Romadhon, Bagus Aulia Mahdi, Afifah Zahra Dzakiyah, Maulana Bagus Adi Cahyono, Narazah Mohd Yusoff

**Affiliations:** 1 Doctoral Program of Medical Science, Faculty of Medicine, Universitas Airlangga, Surabaya 60131, Indonesia; kartikaprahasanti@um-surabaya.ac.id; 2 Department of Physiology, Faculty of Medicine, Muhammadiyah University, 59 Raya Sutorejo Street Mulyorejo, Surabaya 60113, Indonesia; 3 Department of Internal Medicine, Faculty of Medicine, Universitas Airlangga, Surabaya 60131, Indonesia; afifah.zahra.dzakiyah-2023@fk.unair.ac.id; 4 Department of Internal Medicine, Universitas Airlangga Hospital, Surabaya 60115, Indonesia; 5 Department of Physiology, Faculty of Medicine, Universitas Airlangga, Surabaya 60131, Indonesia; lilik_heraw@fk.unair.ac.id; 6 Institute of Tropical Disease, Universitas Airlangga, Surabaya 60115, Indonesia; 7 Department of Internal Medicine, Faculty of Medicine, Muhammadiyah University, 59 Raya Sutorejo Street Mulyorejo, Surabaya 60113, Indonesia; bagusauliamahdi@um-surabaya.ac.id; 8 Medical Program, Faculty of Medicine, Universitas Airlangga, Surabaya 60115, Indonesia; maulanabac01@gmail.com; 9 Advanced Medical and Dental Institute, Universiti Sains Malaysia, Bertam, Kapala Batas 13200, Pulau Pinang, Malaysia; narazah@usm.my

**Keywords:** ineffective erythropoiesis, thalassemia, marker, iron overload, anemia

## Abstract

**Background/Objectives**: Ineffective erythropoiesis (IE) is a hallmark of β-thalassemia and contributes to major clinical complications, including severe anemia, extramedullary hematopoiesis, and progressive iron overload. Despite its central role in disease pathophysiology, there is no established biomarker for the reliable identification and monitoring of IE. This systematic review was conducted to evaluate potential serum markers that reflect IE in β-thalassemia. **Methods**: Across seven databases (PubMed, ScienceDirect, Web of Science, SpringerLink, Taylor & Francis, ProQuest, and SAGE), thirteen studies met the eligibility criteria and were analyzed to identify circulating biomarkers associated with IE in β-thalassemia. **Results**: The most consistently reported markers were growth differentiation factor-15 (GDF-15), soluble transferrin receptor (sTfR), erythropoietin (EPO), and erythroferrone (ERFE), all of which demonstrated strong correlations with the degree of IE and erythroid expansion. Additional markers, including circulating cell-free DNA (cfDNA), CA15.3, hepcidin, ferritin, and phosphatidylserine (PS)-exposed red blood cells, were also found to be elevated, reflecting increased erythroid turnover, apoptosis, and secondary iron dysregulation. These findings suggest that while individual markers capture different aspects of IE, their combined evaluation may provide a more comprehensive picture of disease burden. **Conclusions**: IE represents the central pathophysiological driver of β-thalassemia and is closely linked to disease complications. Early detection through circulating biomarkers offers the potential for timely identification of high-risk patients, monitoring of therapeutic responses, and prognostication. Although current evidence highlights GDF-15, sTfR, ERFE, and EPO as the most promising candidates, further validation in larger, longitudinal cohorts is required before clinical implementation.

## 1. Introduction

β-thalassemia is the most common inherited hemoglobinopathy worldwide [1], particularly in regions across the Mediterranean, Middle East, and Southeast Asia, where carrier prevalence is high [2,3,4,5,6]. This is caused by mutations in the β-globin gene that result in reduced or absent β-globin chain synthesis [1,7,8,9,10]. The consequent imbalance between α- and β-globin chains leads to chronic hemolytic anemia, bone marrow expansion, and a marked state of ineffective erythropoiesis (IE), which remains a central pathophysiological feature of the disease [11,12,13,14,15]. Ineffective erythropoiesis (IE) is defined as the inability to produce a sufficient number of RBCs in the presence of immature erythroid precursors in the bone marrow. Although the introduction of regular blood transfusions and iron chelation therapy has significantly improved survival, complications related to chronic anemia, iron overload, and IE continue to impair quality of life and long-term outcomes in affected patients [16,17,18,19].

Ineffective erythropoiesis contributes not only to severe anemia but also to systemic consequences, including splenomegaly, extramedullary hematopoiesis, bone deformities, and enhanced intestinal iron absorption, further aggravating iron overload [11,15,20,21,22]. Given its pivotal role, quantifying IE has been of considerable interest in recent years. Serum biomarkers, in particular, offer a minimally invasive and widely accessible approach to monitoring disease severity and therapeutic response [15,23]. Several candidates have been proposed, including soluble transferrin receptor (sTfR), growth differentiation factor-15 (GDF-15), erythroferrone (ERFE), and others, each reflecting distinct aspects of erythroid expansion, iron metabolism, or apoptotic signaling [24,25,26].

Nonetheless, the clinical interpretation of these serum markers is complicated by variability in assay methods, overlap with other pathophysiological processes, and inconsistent correlations with disease severity or treatment outcomes. Unlike conventional hematological indices, serum markers may provide mechanistic insights into the biology of IE, but their diagnostic and prognostic value remains uncertain [13,27]. Therefore, a systematic evaluation of serum biomarkers of IE in β-thalassemia is warranted. The aim of this review is to synthesize current evidence on the utility of serum-based markers in assessing ineffective erythropoiesis, to highlight their potential role in clinical practice, and to identify gaps that should be addressed in future research.

## 2. Materials and Methods

This study was conducted according to Preferred Reporting Items for Systematic Reviews and Meta-Analyses (PRISMA). The review protocol was not registered in any database (e.g., PROSPERO). However, the methods were defined a priori and followed throughout the review process.

### 2.1. Literature Search

**Figure 1:**
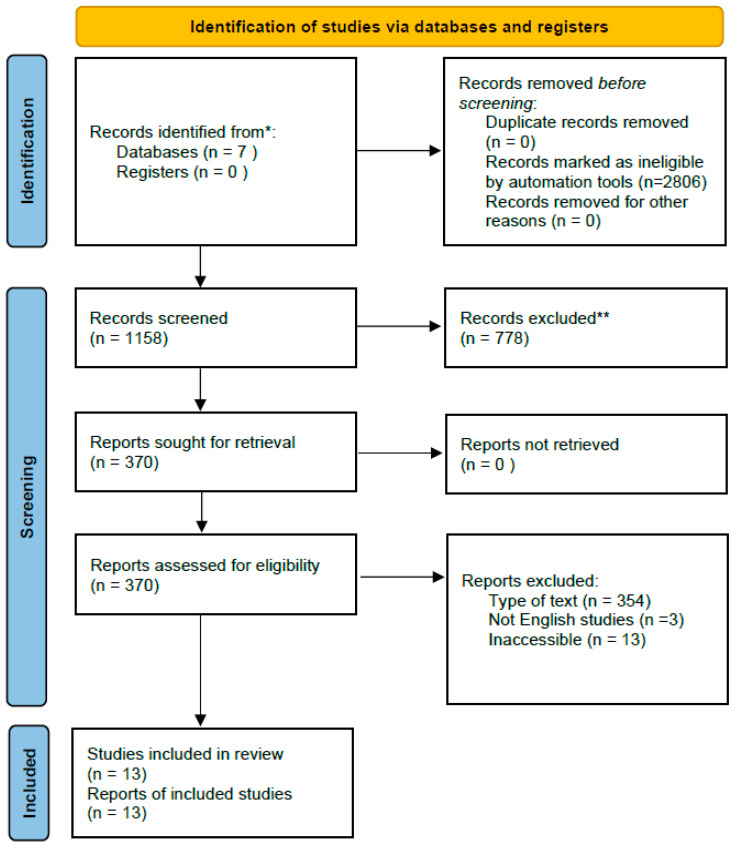
PRISMA flow chart. * Records from each database or register are reported separately where feasible. ** Records were excluded by a human and automation tools.

A comprehensive search of the literature was performed using the seven online databases PubMed, ScienceDirect, Web of Science, SpringerLink, Taylor & Francis, ProQuest, and SAGE. We used the following search terms: (“beta-thalassemia” OR “β-thalassemia” OR thalassemia) AND (“ineffective erythropoiesis” OR “erythroid expansion” OR “erythropoietic activity”) from the randomized control trial search method of the Cochrane Collaboration. Our search approach only included human studies from 2015 to 2025. Each database had a preliminary search in July and a final search on 14 August 2025. The PRISMA flow for a literature search is shown in Figure 1. The PRISMA checklist for this systematic review is available in the Supplementary Materials.

### 2.2. Eligibility and Study Selection

All randomized control trials, cohort studies, case–control studies, and cross-sectional studies reporting IE markers in patients with β-thalassemia. Only English-language publications are included. Duplicate research and full-text studies that could not be recovered were removed. The search was conducted by authors PZR and MBAC. After independently screening the titles and abstracts, three authors (PZR, MBAC, and BAM) obtained the full text of any papers that met the inclusion criteria. Both authors looked at the full-text papers’ eligibility. During the selection process, duplicate studies were removed, and the abstract and full text were examined using the Mendeley application, a free online reference management tool.

### 2.3. Data Extraction

Data extraction was conducted by two reviewers (MBAC, BAM), with any discrepancies resolved through team consensus. The studies meeting the relevance and eligibility criteria were extracted using a pre-designed Excel table. The collected data comprised (1) a summary of the included studies, detailing methodological information regarding the site, sample, interventions, outcomes, and results, and (2) a potential IE marker in β-thalassemia.

## 3. Results

Thirteen studies assessing markers of ineffective erythropoiesis in β-thalassemia were screened and obtained. We summarize these findings in Table 1 and Table 2. Among the included studies, 10 reported significantly elevated GDF-15 levels [24,25,26,28,29,30,31,32,33,34] in patients with transfusion-dependent and non-transfusion-dependent β-thalassemia, with positive correlations to transfusion index and serum ferritin. Eight studies demonstrated increased sTfR concentrations, reflecting heightened erythropoietic activity [25,26,28,29,31,33,35,36]. Two studies identified ERFE as a key mediator suppressing hepcidin, thereby contributing to iron overload [24,37]. In addition, seven studies reported markedly higher EPO levels, consistent with chronic anemia and marrow hyperplasia [24,25,26,29,31,32,36]. One study suggested that circulating cfDNA may serve as a novel marker of erythroid apoptosis [31]. Elevated CA15.3 levels were described in a study conducted by Tavazzi, possibly reflecting expanded erythroid mass [31]. Seven studies consistently reported suppressed hepcidin relative to iron burden [24,25,26,29,34,36,37], whereas nine studies confirmed increased serum ferritin [24,25,26,29,30,34,35,36,37], indicating concomitant iron overload. Finally, two studies described enrichment of PS-exposed RBCs, associated with ineffective erythropoiesis and shortened red cell survival [28,33]. Collectively, these markers (GDF-15, sTfR, ERFE, EPO, cfDNA, CA15.3, hepcidin, ferritin, and PS-exposed RBCs) highlight the multifactorial pathways underlying ineffective erythropoiesis in β-thalassemia and their link to iron dysregulation.

## 4. Discussion

### 4.1. IE

**Figure 2:**
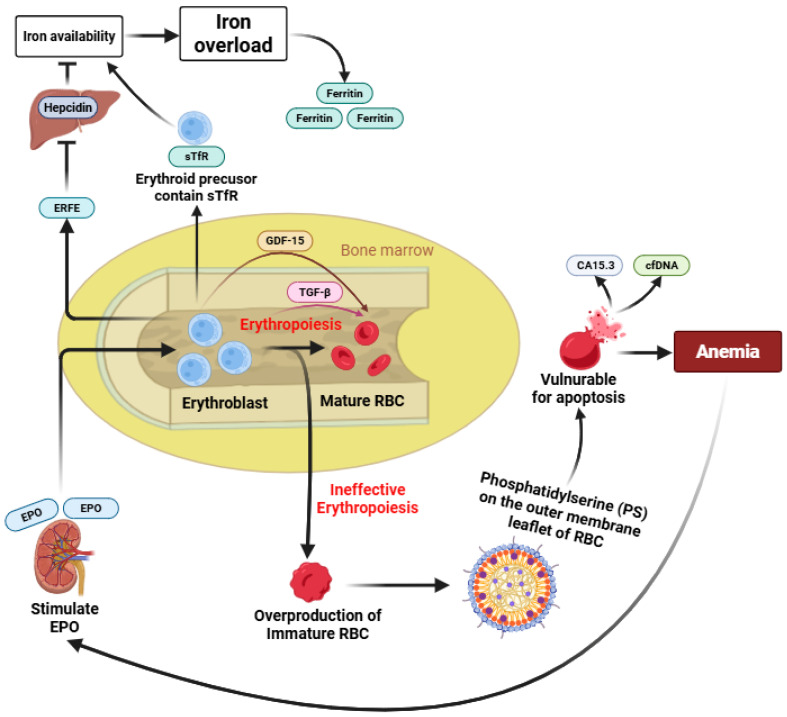
Schematic overview of biomarker regulation in ineffective erythropoiesis.

Ineffective erythropoiesis is a pathological condition in which the bone marrow produces an excessive number of erythroid precursors, but most of these cells undergo premature apoptosis before maturing into functional red blood cells. It results from a complex interaction between molecular mechanisms within the bone marrow and regulatory signals from organs such as the liver, spleen, and gut [11]. In β-thalassaemia, this process is driven largely by the imbalance of globin chains, particularly the excess of α-globin, which triggers apoptosis of developing erythroblasts [8]. The inability to generate adequate mature red blood cells despite increased erythropoietic activity leads to anemia, extramedullary hematopoiesis, splenomegaly, and secondary systemic iron overload [12]. Despite growing interest in ineffective erythropoiesis, its underlying mechanisms remain incompletely defined, and no single biomarker has been established as a definitive or gold-standard indicator. The available biomarkers reflect different and partially overlapping aspects of erythroid expansion, maturation failure, iron dysregulation, and premature erythrocyte clearance. To integrate these heterogeneous pathways and to provide a conceptual framework for their interrelationships, we present a schematic overview summarizing the regulation and interaction of the nine biomarkers associated with ineffective erythropoiesis in β-thalassemia (Figure 2).

Under normal conditions, erythropoiesis results in the maturation of erythroblasts into functional mature RBCs [1]. In β-thalassemia, reduced or absent β-globin chain synthesis leads to IE, a complex process that remains incompletely understood, characterized by erythroid expansion with impaired maturation and increased intramedullary destruction [8]. As a consequence, immature and structurally abnormal erythrocytes accumulate and exhibit phosphatidylserine (PS) externalization on the cell membrane, triggering eryptosis and accelerated clearance, thereby contributing directly to anemia [33]. Enhanced apoptosis and intramedullary destruction of erythroid precursors are associated with increased release of circulating biomarkers such as CA15.3 and cfDNA [31,38]. Chronic anemia stimulates renal EPO production, which drives expansion of erythroid progenitors and induces ERFE secretion from erythroblasts [39]. Elevated ERFE suppresses hepatic hepcidin expression, leading to increased iron availability in the circulation. Concurrently, expanded erythroid mass expresses higher levels of sTfR, further enhancing iron uptake [40]. The combined effects of increased iron absorption and utilization result in progressive iron overload, reflected by elevated serum ferritin levels. In parallel, erythroid stress and expanded but ineffective erythropoiesis promote increased expression of GDF-15 across early to late stages of erythroid differentiation, with additional involvement of TGF-β signaling during late erythroid maturation [32]. Together, these interconnected pathways illustrate how alterations in multiple biomarkers reflect different yet overlapping aspects of ineffective erythropoiesis, iron dysregulation, and premature erythrocyte death in β-thalassemia (Figure 2).

Normal erythropoiesis results in the maturation of erythroblasts into functional red blood cells (RBCs). In β-thalassemia, impaired β-globin synthesis leads to ineffective erythropoiesis, characterized by expanded but dysfunctional erythroid precursors and accumulation of immature RBCs. Premature phosphatidylserine (PS) exposure on immature erythrocytes promotes eryptosis and accelerated clearance, contributing to anemia. Enhanced erythroid apoptosis is associated with increased release of CA15.3 and cell-free DNA (cfDNA). Chronic anemia induces erythropoietin (EPO) production, stimulating erythroid expansion and erythroferrone (ERFE) secretion, which suppresses hepcidin and increases iron availability. Elevated soluble transferrin receptor (sTfR) further enhances iron uptake, leading to iron overload reflected by increased ferritin levels. Erythroid stress also induces growth differentiation factor-15 (GDF-15) across erythroid differentiation stages, highlighting the interconnected regulation of biomarkers in ineffective erythropoiesis.

### 4.2. GDF-15

Growth differentiation factor-15 (GDF-15) is a stress-induced cytokine [41] produced primarily by erythroid precursors during ineffective erythropoiesis [42]. During the early to late stages of erythroid differentiation, GDF15 levels rise. It has an inhibitory effect on the development of erythroid cells in vitro, affecting their maturation and apoptosis processes. Elevated circulating GDF-15 levels have been consistently observed in β-thalassemia patients, particularly in those receiving regular transfusions. GDF-15 may be considered to be a biomarker for evaluating erythropoiesis activity, both in qualitative and quantitative ways [43]. GDF-15 plays a key role in iron metabolism by suppressing hepcidin expression, thereby promoting increased intestinal iron absorption despite systemic iron overload. In TDT patients, a mild but highly consistent negative correlation was observed between Hb and GDF15 levels. High GDF-15 levels in transfusion-dependent β-thalassemia are influenced not only by erythropoietic stress but also by additional mechanisms such as tissue hypoxia and iron-mediated oxidative stress, and levels may indeed vary depending on the degree of transfusion suppression. However, moderately increased GDF-15 in anemia of unknown origin was not significantly correlated with hepcidin expression. Thus, the relationship between GDF-15 and hepcidin in non-anemic patients remains positive [44]. Several studies have demonstrated a positive correlation between GDF-15 levels, transfusion index, and serum ferritin, highlighting its potential utility as a biomarker linking ineffective erythropoiesis with dysregulated iron homeostasis [25,30,34].

### 4.3. sTfR

Soluble transferrin receptor (sTfR) is an extracellular domain part of the transferrin receptor (TfR), and its concentration is proportional to the amount of TfR in the total body. sTfR reflects the degree of erythropoietic activity and iron demand at the cellular level, regardless of iron status. Therefore, sTfR will increase in increased oxygen demand conditions, although without anemia [45]. sTfR is one of the most significant markers associated with chronic disease, despite anemia, such as its association with a higher risk of diabetes mellitus (DM) in overweight and obese subjects. In β-thalassemia, sTfR levels are markedly elevated due to expanded yet ineffective erythropoiesis [46]. Unlike ferritin, which reflects iron storage, sTfR is less influenced by inflammation and more directly associated with the severity of anemia and marrow drive [47]. Elevated sTfR has been consistently reported in transfusion-dependent and non-transfusion-dependent thalassemia, reinforcing its role as a reliable surrogate for erythropoietic burden [25,28,29].

### 4.4. ERFE

Erythroferrone (ERFE) is a glycoprotein hormone with the primary function of Erythroferrone as a negative regulator of hepcidin, which is central to iron homeostasis. ERFE is a prime candidate to exert a similar role in dysfunctional erythropoiesis because it is a hormone secreted by erythroblasts in response to erythropoietin stimulation, which is associated with iron overload. ERFE can influence hepcidin expression both in long-term condition such as ineffective erythropoiesis, and acute events, such as erythropoietin (EPO) injection or phlebotomy [48]. It acts on hepatocytes as a potent suppressor of hepcidin production, thereby enhancing dietary iron absorption and mobilization from stores [49]. In β-thalassemia, persistent elevation of ERFE contributes to the inappropriate downregulation of hepcidin, despite systemic iron overload [50,51]. Experimental and clinical studies confirm ERFE as a pivotal mediator linking ineffective erythropoiesis with pathological iron accumulation [24].

### 4.5. EPO

Erythropoietin (EPO) is a renal glycoprotein hormone that stimulates erythroid progenitor proliferation and differentiation. EPO is produced by the liver in the fetus; thus, the kidney is the main producer of EPO after birth [52]. In β-thalassemia, chronic anemia and tissue hypoxia drive persistently elevated EPO levels. In patients with chronic anemia and preserved renal function, EPO levels are typically elevated as a physiological response to tissue hypoxia. In conditions characterized by ineffective erythropoiesis, such as β-thalassemia, persistently increased EPO reflects sustained erythropoietic drive and correlates with clinical manifestations of erythroid expansion, including bone marrow hyperplasia and extramedullary hematopoiesis. However, elevated EPO should be interpreted as an upstream driver of erythropoietic stress rather than a direct or specific biomarker of ineffective erythropoiesis. By binding to the EPO receptor (EPOr) on the surface of erythroid cells, EPO allows the proliferation and survival of erythroid cells and determines the production of erythrocytes at homeostasis and under hypoxic conditions. Although this enhances erythroid expansion, the imbalance between proliferation and maturation results in ineffective erythropoiesis [39,53]. Elevated EPO levels have been positively correlated with marrow hyperplasia and splenomegaly, underscoring its role in the compensatory but maladaptive response in β-thalassemia [12].

### 4.6. cfDNA

Circulating cell-free DNA (cfDNA) is released from apoptotic and necrotic cells, including erythroid precursors undergoing intramedullary destruction [38]. Recent studies have reported increased cfDNA levels in β-thalassemia patients, suggesting it as a potential biomarker of ineffective erythropoiesis [31]. In β-thalassemia, elevated cfDNA levels are thought to originate, at least in part, from enhanced apoptosis of erythroid precursors within the bone marrow, a hallmark of ineffective erythropoiesis. While its specificity is limited, cfDNA may serve as an early indicator of erythroid apoptosis and a potential non-invasive marker to monitor disease activity and discrimination between different types of anemia compared with reticulocytic count [54].

### 4.7. CA15.3

Cancer antigen 15.3 (CA15.3), a mucin-like glycoprotein traditionally used in oncology, has also been implicated in thalassemia as a surrogate of erythroid mass expansion. In hematological diseases associated with ineffective erythropoiesis, such as thalassemia and sickle cell, CA15.3 concentration can increase to 6 times higher than normal [55]. Elevated CA15.3 levels in β-thalassemia patients have been attributed to excessive erythroid turnover rather than malignancy. Although evidence remains limited, early studies suggest CA15.3 may complement established markers in assessing ineffective erythropoiesis [31].

### 4.8. Hepcidin

Hepcidin is the master regulator of systemic iron homeostasis, controlling both intestinal absorption and macrophage iron release. By switching off ferroportin in enterocytes and macrophages, hepcidin exerts fine control over both iron absorption and its distribution among tissues. Hepcidin expression is downregulated by low iron status and active erythropoiesis and upregulated by iron overload, infection and inflammation [40]. In β-thalassemia, hepcidin levels are inappropriately suppressed relative to iron burden, largely due to the influence of erythroid regulators such as ERFE. Alterations in hepcidin levels in β-thalassemia largely occur downstream of erythroid-derived signals, including ERFE. As ERFE measurement is not routinely available in clinical practice, the combined assessment of hepcidin and sTfR may provide a pragmatic approach to indirectly evaluate erythropoietic drive and its effects on iron regulation [25,56,57]. This downregulation drives pathological iron loading, even in the absence of transfusion. Numerous studies have confirmed the hepcidin–erythropoiesis axis as central to the pathophysiology of iron overload in thalassemia [58,59].

### 4.9. Ferritin

Ferritin is an iron storage protein, and measurement in the plasma or serum reflects iron stores in healthy individuals. Low ferritin indicates iron deficiency, while elevated ferritin indicates iron overload. However, ferritin is also an acute-phase protein that can be elevated in inflammation and infection. Therefore, the coexistence of iron deficiency and inflammation can result in relatively higher serum ferritin compared to similar body iron stores without inflammation, and it requires serial measurements. Serum ferritin remains the most widely used marker of body iron stores [60]. In β-thalassemia, ferritin levels are consistently elevated, reflecting both transfusional iron input and increased absorption secondary to hepcidin suppression. Ferritin level is higher in β-thalassemia major with dependent transfusion than in thalassemia intermediate [61]. While ferritin is a useful screening tool, its levels may be influenced by inflammation and liver pathology, necessitating complementary measures such as MRI-based iron quantification. However, ferritin levels in women are normally lower than in men [62]. Nevertheless, ferritin continues to be a practical surrogate for monitoring iron overload in both clinical and research settings, although it cannot predict bone marrow Fe content [63].

### 4.10. PS-Exposed RBCs

Phosphatidylserine (PS)-exposed red blood cells represent a subpopulation of circulating erythrocytes with externalized PS on the outer leaflet of the plasma membrane [64]. Phosphatidylserine exposure on red blood cells represents a key pathophysiological event in β-thalassemia. During ineffective erythropoiesis, immature and structurally abnormal erythrocytes undergo premature phosphatidylserine externalization, which acts as a signal for eryptosis and macrophage-mediated clearance. This process leads to reduced erythrocyte lifespan and directly contributes to anemia, highlighting PS-exposed RBCs as a particularly promising biomarker linking erythroid membrane pathology to clinical disease manifestations. In β-thalassemia, excess ineffective erythropoiesis and oxidative stress lead to increased PS exposure, which promotes erythrophagocytosis and reduces red cell survival. Elevated PS-exposed RBCs have been associated with anemia severity, hemolysis, and hypercoagulability in β-thalassemia, suggesting a multifactorial role in disease complications [33,65].

## 5. Conclusions

This systematic review demonstrates that multiple biomarkers, including GDF-15, sTfR, ERFE, EPO, cfDNA, CA15.3, hepcidin, ferritin, and PS-exposed RBCs, provide complementary insights into the mechanisms of ineffective erythropoiesis in β-thalassemia. Their consistent associations with iron overload and disordered erythroid expansion highlight their potential utility not only as disease monitoring tools but also as therapeutic targets to improve patient management. Although several biomarkers related to ineffective erythropoiesis have been proposed in β-thalassemia, significant knowledge gaps remain. Importantly, robust prospective clinical trials validating these biomarkers are still lacking. Future research should focus on defining cut-off values, understanding biomarker dynamics in response to treatment (transfusion, chelation, novel agents), and evaluating their prognostic significance across different disease phenotypes. Addressing these evidence gaps will support the development of clinically reliable biomarker-based tools that could optimize monitoring, personalize therapy, reduce complications such as iron overload and splenomegaly, and, ultimately, improve quality of life in people living with β-thalassemia.

## Figures and Tables

**Table 1:** IE marker reported from study of β-thalassemia.

No.	Authors, Year	Study Type	Population (Number of Patients)	Marker Reported
1.	Guimarães, 2015 [29]	Cross-sectional	Control group (28)	Hepcidin
			α-thalassemia carriers (14)	ferritin
			β-thalassemia carriers (20)	GDF-15
			β-thalassemia major (27)	EPO
				sTfR
2.	Tantawy, 2015 [30]	Cross-sectional	β-thalassemia intermedia (35)	ferritin
			Control group (35)	GDF-15
3.	Ragab, 2015 [35]	Cross-sectional	Control group (20)	ferritin
			β-thalassemia intermedia (20)	sTfR
			β-thalassemia major (22)	haptoglobin
4.	Sulovska, 2016 [26]	Cohort	β-thalassemia intermedia (10)	GDF-15
			β-thalassemia major (1)	sTfR
			α-thalassemia intermedia (2)	EPO
				Hepcidin
				ferritin
5.	Tavazzi, 2018 [31]	Cohort	Non-transfusion-dependent β-thalassemia (49)	cfDNA
				EPO
				sTfR
				GDF-15
				CA15.3
6.	Huang, 2019 [25]	Cross-sectional	Non-transfusion-dependent thalassemia (192)	GDF-15
			Transfusion-dependent thalassemia (94)	EPO
				sTfR
				Hepcidin
				Ferritin
7.	Smesam, 2020 [37]	Cross-sectional	β-thalassemia major (60)	ERFE
			Control group (30)	Hepcidin
				Ferritin
				transferin
8.	Khairullah, 2021 [36]	Cross-sectional	β-non-transfusion-dependent thalassemia (11)	EPO
			β-transfusion-dependent thalassemia (9)	sTfR
				Hepcidin
				Ferritin
9.	Ozturk, 2021 [24]	Cross-sectional	Thalassemia major (21)	EPO
			Thalassemia intermedia (20)	ERFE
			Thalassemia trait (20)	GDF-15
			Control group (22)	Hepcidin
				Ferritin
10.	Yousif, 2022 [32]	Cross-sectional	β-thalassemia major (18)	TGF-β1
			β-thalassemia intermedia (17)	GDF-15
			Control group (20)	EPO
11.	Chansai, 2022 [33]	Cross-sectional	β-non-transfusion-dependent thalassemia (40)	PS-exposed RBCs
			β-transfusion-dependent thalassemia (48)	GDF-15
				sTfR
12.	Chansai, 2022 [28]	Cross-sectional	β-non-transfusion-dependent thalassemia (40)	PS-exposed RBCs
			β-transfusion-dependent thalassemia (48)	GDF-15
				sTfR
13.	Meena, 2023 [34]	Cross-sectional	β-thalassemia major (39)	GDF-15
			Control group (33)	Hepcidin
				Ferritin

**Table 2:** Result and conclusion from study of IE markers in β-thalassemia.

No.	Authors, Year	Results–Conclusions
1.	Guimarães, 2015 [29]	In β-thalassemia carriers, elevated erythropoietic activity is indicated by increased serum levels of sTfR and EPO. In patients undergoing bone marrow transplantation (BMT), there are also increased levels of sTfR, EPO, sTfR/log ferritin, and 1515. This suggests that, despite receiving RBC transfusions, expanded erythropoiesis is likely occurring but remains ineffective.
2.	Tantawy, 2015 [30]	GDF-15 levels are markedly increased in patients with thalassemia intermedia, especially among those undergoing regular transfusions. Levels exhibit a positive correlation with transfusion index and serum ferritin. GDF-15 contributes to iron overload by suppressing hepcidin, even in the presence of adequate erythropoiesis. Ineffective erythropoiesis is proposed to lead to iron overloading by inhibiting the necessary increase in hepcidin expression.
3.	Ragab, 2015 [35]	Haptoglobin (Hp) depletion in patients with thalassemia results from hemolysis and ineffective erythropoiesis (IE). The serum Hp level was significantly lower in TM children compared to TI children and exhibited a notable inverse correlation with the degree of erythropoietic activity, as measured by sTfR, which was identified as the sole predictor of its level.
4.	Sulovska, 2016 [26]	The inhibition of hepcidin by erythropoietin (EPO) occurs indirectly and necessitates active erythropoiesis in the bone marrow. Additionally, growth differentiation factor 15 (GDF-15) is regarded as a marker of ineffective erythropoiesis rather than the primary suppressor of hepcidin. Increased levels of sTfR and GDF-15 suggest heightened yet ineffective erythropoiesis within the bone marrow.
5.	Tavazzi, 2018 [31]	The concentration of cfDNA exhibited a correlation with parameters of iron deficiency erythropoiesis (IE), including erythroblast count and soluble transferrin receptor (sTfR), in addition to indirect markers such as GDF-15 and CA15.3.
6.	Huang, 2019 [25]	Thalassemia patients exhibited iron overload, decreased hepcidin levels, and an increased degree of ineffective erythropoiesis. Patients with transfusion-dependent thalassemia (TDT) exhibited significantly elevated levels of GDF-15 and EPO in comparison to those with non-transfusion-dependent thalassemia (NTDT). Patients with iron overload exhibited elevated levels of EPO, GDF-15, SF, and sTfR in comparison to those without iron overload. Hepcidin levels exhibited a stronger correlation with ineffective erythropoiesis than with iron overload.
7.	Smesam, 2020 [37]	In thalassemia, there is an increase in erythropoiesis that is inefficient, leading to the up-regulation of ERFE and the down-regulation of hepcidin.
8.	Khairullah, 2021 [36]	The association between ineffective erythropoiesis and iron overload is evidenced by the positive correlation of both EPO and sTfR with iron loading, as indicated by serum ferritin levels.
9.	Ozturk, 2021 [24]	GDF-15 may serve as a marker for ineffective erythropoiesis in thalassemia; however, ERFE may be more closely associated with this condition.
10.	Yousif, 2022 [32]	Ineffective erythropoiesis results in elevated levels of EPO and GDF-15. The tumor growth factor-β (TGF-β) family exerts a negative regulation on erythrocyte differentiation and maturation, whereas EPO serves as a regulator of early-stage erythropoiesis.
11.	Chansai, 2022 [28]	GDF-15 may serve as a biomarker indicative of ineffective erythropoiesis in patients with thalassemia, whereas sTfR may lack specificity for ineffective erythropoiesis and is regarded as a general biomarker of erythropoietic activity.
12.	Chansai, 2022 [33]	Extramedullary hematopoiesis occurs more frequently in individuals with β-thalassemia compared to those with α-thalassemia. Among the biomarkers of ineffective erythropoiesis, PS-exposed RBCs demonstrated a modest correlation with extramedullary hematopoiesis.
13.	Meena, 2023 [34]	GDF-15 concentration reflects competing influences of anemia (ineffective erythropoiesis) and iron overload.

## Data Availability

Not applicable.

## Supplementary Materials

The following supporting information can be downloaded at: https://www.mdpi.com/article/10.3390/jcm15010308/s1, PRISMA Checklist. Reference [66] is cited in Supplementary Materials.

## Abbreviations

The following abbreviations are used in this manuscript:

BMT	Bone marrow transplantation
cfDNA	Cell-free DNA
EPO	Erythropoietin
ERFE	Erythroferrone
GDF-15	Growth differentiation factor-15
Hp	Haptoglobin
IE	Ineffective erythropoiesis
NTDT	Non-transfusion-dependent thalassemia
PRISMA	Preferred reporting items for systematic reviews and meta-analyses
PS	Phosphatidylserine
RBCs	Red blood cells
sTfR	Soluble transferrin receptor
TDT	Transfusion-dependent thalassemia
TGF-β	Tumor growth factor-β
